# Elevation of Chemosensitivity of Lung Adenocarcinoma A549 Spheroid Cells by Claudin-2 Knockdown through Activation of Glucose Transport and Inhibition of Nrf2 Signal

**DOI:** 10.3390/ijms22126582

**Published:** 2021-06-19

**Authors:** Ayaka Ito, Haruka Nasako, Risa Akizuki, Yui Takashina, Hiroaki Eguchi, Toshiyuki Matsunaga, Yuta Yoshino, Satoshi Endo, Akira Ikari

**Affiliations:** 1 Laboratory of Biochemistry, Department of Biopharmaceutical Sciences, Gifu Pharmaceutical University, Gifu 501-1196, Japan; 175012@gifu-pu.ac.jp (A.I.); 155050@gifu-pu.ac.jp (H.N.); ri.aki1208@gmail.com (R.A.); 145037@gifu-pu.ac.jp (Y.T.); 146008@gifu-pu.ac.jp (H.E.); yoshino-yu@gifu-pu.ac.jp (Y.Y.); sendo@gifu-pu.ac.jp (S.E.); 2 Education Center of Green Pharmaceutical Sciences, Gifu Pharmaceutical University, Gifu 502-8585, Japan; matsunagat@gifu-pu.ac.jp

**Keywords:** claudin, lung adenocarcinoma, chemoresistance, glucose

## Abstract

Claudin-2 (CLDN2), a tight junctional protein, is involved in the chemoresistance in a three-dimensional spheroid culture model of human lung adenocarcinoma A549 cells. However, the mechanism has not been fully clarified. We found that the knockdown of CLDN2 expression by siRNA in the spheroid reduces the expression of glucose transporters and metabolic enzymes. In a two-dimensional culture model, the expression of these proteins was increased by glucose deprivation or fasentin, an inhibitor of glucose transporter. In addition, the expression levels of nuclear factor erythroid 2-related factor 2 (Nrf2) and antioxidant enzymes including heme oxygenase-1, NAD(P)H:quinone oxidoreductase-1, and a glutamate–cysteine ligase modifier subunit were increased by fasentin. The fluorescence intensities of JC-1, a probe of mitochondrial membrane potential, and MitoROS 580, a probe of mitochondrial superoxide production, were increased by fasentin. These results suggest that mitochondrial production of reactive oxygen species is increased by glucose deficiency. The knockdown of CLDN2 enhanced the flux of 2-deoxy-2-[(7-nitro-2,1,3-benzoxadiazol-4-yl)amino]-D-glucose (2-NBDG), a fluorescent deoxyglucose derivative, in a transwell assay, and the accumulation of glucose and 2-NBDG in spheroid cells. The expression of Nrf2 was decreased by CLDN2 knockdown, which was inhibited by fasentin and sulforaphane, a typical Nrf2 activator, in spheroid cells. The sensitivity of spheroid cells to doxorubicin, an anthracycline antitumor antibiotic, was enhanced by CLDN2 knockdown, which was inhibited by fasentin and sulforaphane. We suggest that CLDN2 induces chemoresistance in spheroid cells mediated through the inhibition of glucose transport and activation of the Nrf2 signal.

## 1. Introduction

A solid tumor microenvironment is created by cancer cells, extracellular matrix, and non-cancer cells such as fibroblasts, macrophages, and vascular endothelial cells in the body [1]. Numerous immature blood vessels are formed in the microenvironment. Therefore, the cells in the central region of the microenvironment are commonly exposed to hypoxic, oxidative and nutritional stress conditions. These stress conditions correlate with therapeutic resistance and metastasis, suggesting that stress adaptation is a critical survival advantage for cancer cells. Hypoxia-inducible factor-1 (HIF-1) and nuclear factor-erythroid 2-related factor 2 (Nrf2) play important roles in the hypoxic and oxidative stress responses, respectively. The improvement of stress conditions may be useful to prevent the tumor progression. Thus, the pharmacological targeting of the HIF-1 and Nrf2 signaling pathways was considered as a new strategy for cancer therapy in recent years [2,3].

Extracellular glucose is transported into cancer cells through specific glucose transporter proteins including the sodium-dependent glucose transporters (SGLTs) family and the independent glucose transporters (GLUTs) family. The SGLTs are active cotransporters, whereas the GLUTs are passive transporters. The GLUTs family consists of fourteen members (GLUT1-14) and is divided into three subclasses (class I–III) based on sequence similarities [4]. GLUT1-4 contained in class I are the most well-characterized examples of this family. Both GLUT1 and GLUT3 expressions are upregulated in various solid tumors including lung, pancreas, colon, and so on [5,6]. After glucose is transported into cells, it undergoes metabolization by using several pathways including the glycolysis and pentose phosphate pathway. The Glycolysis pathway serves to produce adenosine triphosphate (ATP), a high-energy storage molecule. Cancer cells specifically utilize this process rather than mitochondrial oxidative phosphorylation, not only in anaerobic but also in an aerobic environment, which is the so-called Warburg effect [7]. In contrast, mitochondria may have roles such as generation of reactive oxygen species (ROS) and regulation of cell death in addition to ATP production [8].

Tight junctions (TJs) formed between the opposed epithelial cells play a role to regulate paracellular permeability to ions and small molecules. The function of TJs is mainly controlled by claudins (CLDNs), four transmembrane proteins [9]. CLDNs constitute a family of over twenty subtypes [10]. Each subtype is expressed with different patterns in the tissues of the whole body. The abnormal expression of CLDN subtypes was reported in various solid tumors. So far, we have reported that CLDN2 is highly expressed in human lung adenocarcinoma tissues and cell lines [11]. The knockdown of CLDN2 expression by siRNA induces the inhibition of proliferation and migration in human lung adenocarcinoma A549 cells [12,13]. In addition, the sensitivity to anticancer drugs including cisplatin and doxorubicin (DXR) is enhanced by CLDN2 knockdown in the spheroids [14], an in vitro model of non-vascularized early tumor growth. CLDN2 can serve to retain cyclin D1, a regulator of cell-cycle transition through the G1 phase, in the nucleus, resulting in the promotion of cell cycle progression. In contrast, the chemoresistance mechanism by increased CLDN2 expression has not been completely elucidated yet.

In the present study, we found that the expression levels of glucose transporters and metabolic enzymes in A549 spheroid cells are higher than those in the two-dimensional (2D) culture model. The effect of CLDN2 on the expression of these transporters and enzymes was investigated by real-time polymerase chain reaction (PCR) and Western blotting analyses. The barrier function of CLDN2 was estimated by transepithelial electrical resistance (TER) and permeability to a fluorescent glucose probe. Mitochondria activity was examined using JC-1, a fluorescent indicator for measuring the membrane potential of mitochondria, and a fluorescent indicator of mitochondrial ROS production. Our results indicate that CLDN2 may be involved in the ROS generation mediated by the activation of mitochondria in A549 spheroid cells.

## 2. Results

### 2.1. Increase in the Expression of Glucose Transporters and Metabolic Enzymes in A549 Spheroid Cells

**Figure 1:**
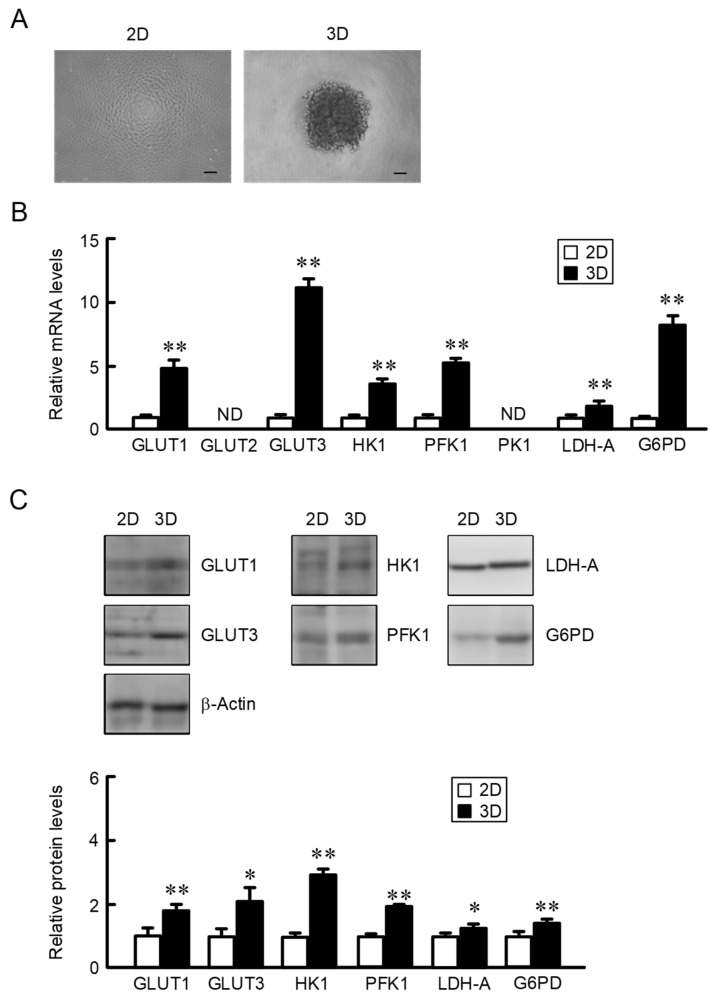
Elevation of glucose transporters and metabolic enzymes by 3D culture. A549 cells were cultured on 6 cm flat dishes (2D) or 96-well round-bottomed plates (3D) for 4 days. (**A**) Representative images of 2D monolayer and 3D spheroid cells. Scale bar indicates 100 μm. (**B**) Real-time PCR was performed using primer pairs as listed in Table 1. The mRNA levels are represented relative to the values in 2D. ND means under the limit of detection. (**C**) Western blotting was performed using antibodies as listed in Table 2. The protein levels are represented relative to the values in 2D. *n* = 3–4. ** *p* < 0.01 and * *p* < 0.05 compared with 2D.

Glucose is transported into the cancer cells mediated through glucose transporters such as GLUTs. The expression levels of GLUTs were compared using 2D monolayer culture cells, which were maintained under confluent conditions, and 3D spheroids, whose circumference was 1860.0 ± 12.9 μm (*n* = 32). The mRNA levels of GLUT1 and GLUT3 in A549 spheroid cells were higher than those in 2D monolayer culture cells (Figure 1A). The expression of GLUT2 was not detected under our experimental conditions. The uptaken glucose is metabolized by the glycolysis pathway which contains a series of reactions for converting glucose into lactate. The rate-limiting reactions are catalyzed by hexokinase 1 (HK1), phosphofructokinase 1 (PFK1), and pyruvate kinase 1 (PK1). The mRNA levels of HK1 and PFK1 in A549 spheroid cells were higher than those in 2D cells. The expression of PK1 was not detected under our experimental conditions. The mRNA levels of lactate dehydrogenase-A (LDH-A), which executed the final step of anaerobic lactate production, and glucose-6-phosphate dehydrogenase (G6PD), which catalyzes the rate-limiting step in the pentose phosphate pathway, were also increased in spheroid cells compared with 2D cells. Western blot measurement showed that the protein levels of GLUT1, GLUT3, HK1, PFK1, LDH-A, and G6PD were increased in spheroid cells (Figure 1B). These results are similar to those in real-time PCR measurement.

### 2.2. Comparison of the Expression of HIF-1α and Nrf2 in 2D Monolayer and Three-Dimensional (3D) Spheroid Cells

**Figure 2:**
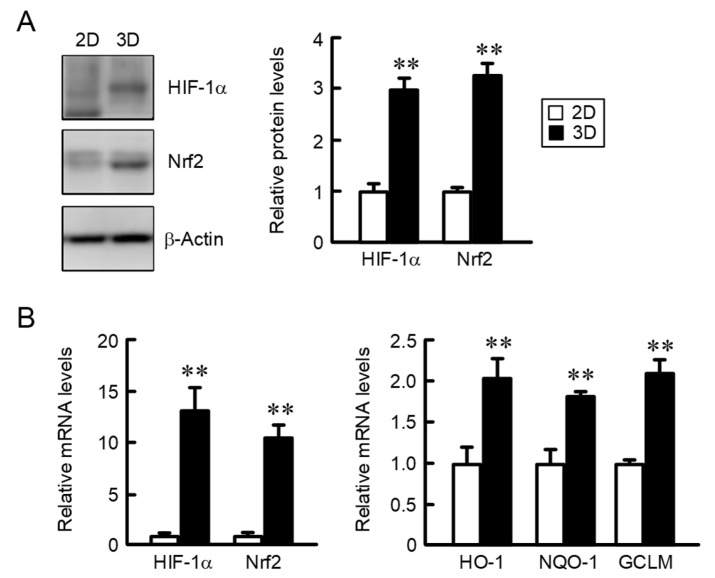
Elevation of HIF-1α and Nrf2 expression by 3D culture. A549 cells were cultured on 6 cm flat dishes (2D) or 96-well round-bottomed plates (3D) for 4 days. (**A**) Western blotting was performed using anti-HIF-1α, anti-Nrf2, and anti-β-actin antibodies. The protein levels are represented relative to the values in 2D. (**B**) Real-time PCR was performed using primer pairs as listed in Table 1. The mRNA levels are represented relative to the values in 2D. *n* = 3–4. ** *p* < 0.01 compared with 2D.

The correlation between GLUT1 expression and hypoxia was reported using human cancer tissues [15]. The cells within the central region of spheroids may be exposed to hypoxic conditions. The protein levels of HIF-1α in 3D spheroid cells were higher than that in the 2D monolayer cells (Figure 2A). In addition, the expression of Nrf2, HIF-1α, and the Nrf2-targeted genes including NAD(P)H: quinone oxidoreductase-1 (NQO1), heme oxygenase-1 (HO-1), and glutamate–cysteine ligase modifier subunit (GCLM) was increased in 3D spheroid cells (Figure 2B). These results indicate that the cells within spheroids may be exposed to hypoxia and oxidative stresses.

### 2.3. Decreases in Nrf2 and the Nrf2-Targeted Genes by CLDN2 Knockdown

**Figure 3:**
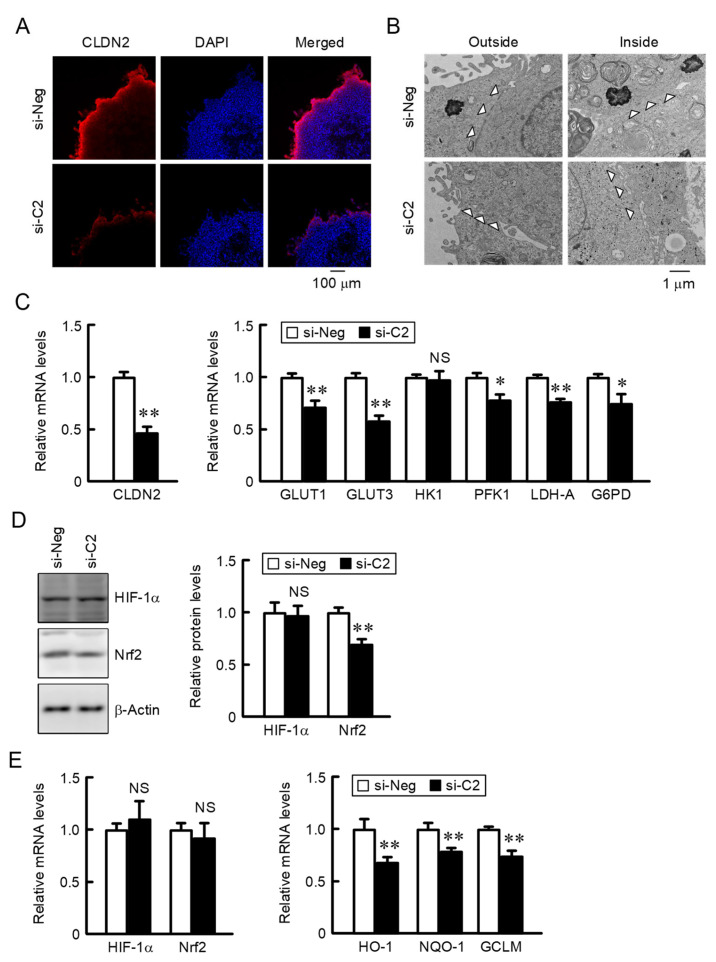
Decrease in glucose transporters and metabolic enzymes by CLDN2 knockdown in 3D spheroid cells. A549 cells transfected with negative (si-Neg) or CLDN2 siRNA (si-C2) were cultured in 96-well round-bottomed plates for 4 days. (**A**) The localization of CLDN2 protein (red) was examined using confocal laser microscopy. The nuclei are stained with DAPI (blue). (**B**) The structure of the tight junction was observed by transmission electron microscope. The kissing points are indicated as arrowheads. (**C**,**E**) Real-time PCR was performed using primer pairs as listed in Table 1. The mRNA levels are represented relative to the values in si-Neg. (**D**) Western blotting was performed using anti-HIF-1α, anti-Nrf2 and anti-β-actin antibodies. The protein levels are represented relative to the values in si-Neg. *n* = 3–4. ** *p* < 0.01 and * *p* < 0.05 compared with si-Neg. NS, *p* > 0.05.

So far, we have reported that CLDN2 contributes to the acquisition of chemoresistance in A549 spheroid cells [14]. Spheroid cells were transfected with siRNA against negative control or CLDN2. After treating with CytoVista 3D Cell Culture Clearing buffer, the spheroid cells were stained with anti-CLDN2 antibody and 4′,6-diamidino-2-phenylindole (DAPI). The strong fluorescence signal of CLDN2 was detected in the outer layer of spheroids, which was reduced by CLDN2 knockdown (Figure 3A). In the electron microscope observation, the TJ structure so-called “kissing-point” was observed at the cell-cell contact region in the outer layer of spheroids. In contrast, the kissing point was little detected in the inside of the spheroid of CLDN2-expressing cells and both the outer and inside of the spheroid of CLDN2 knockdown cells (Figure 3B). These results indicate that CLDN2 may be involved in the formation of TJ in the outer layer of spheroids. DNA microarray analysis showed that the expression levels of 6-phosphofructo-2-kinase/Fructose-2,6-biphosphatase 4 (PFKFB4) and 6-phosphofructokinase (PFKL) genes, which are involved in the glucose metabolization, were downregulated by CLDN2 knockdown (Table 3). Therefore, we investigated the effect of CLDN2 knockdown on glucose transporters and metabolic enzymes by real-time PCR analysis. The mRNA expression of these proteins was decreased by CLDN2 knockdown in spheroid cells (Figure 3C). The expression levels of Nrf2 protein and the Nrf2-targeted genes were decreased by CLDN2 knockdown in spheroid cells (Figure 3D,E). Similarly, the mRNA levels of glucose transporters, metabolic enzymes, and Nrf2-targeted genes were decreased by CLDN2 knockdown in spheroids of human lung adenocarcinoma PC-3 cells (Supplementary Figure S1). These results indicate that CLDN2 may be involved in the development of oxidative stress condition in spheroid cells of lung adenocarcinoma. In addition, CLDN2 knockdown decreased the expression of Nrf2 protein without affecting its mRNA level (Figure 3D), indicating that the protein expression of Nrf2 may be controlled in the post-translational step. Similar results were obtained in PC-3 cells (Supplementary Figure S2). HIF-1α may not be involved in the CLDN2 knockdown response because the expression levels of HIF-1α mRNA and protein were not changed by CLDN2 knockdown in spheroid cells.

### 2.4. Effects of Glucose and Lactate Concentration on the Expression of Glucose Transporters and Metabolic Enzymes in 2D Monolayer and 3D Spheroid Cells

**Figure 4:**
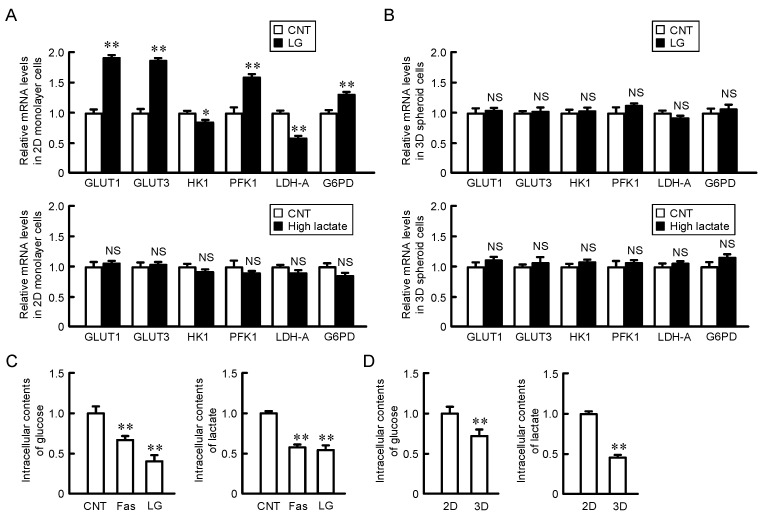
Effects of low glucose and high lactate on the expression of glucose transporters and metabolic enzymes in 2D monolayer and 3D spheroid cells. (**A**) A549 monolayer cells were incubated with the media containing 25 mM glucose (CNT), 5 mM glucose (LG), or 20 mM lactate (High lactate) for 6 h. (**B**) A549 spheroid cells were incubated with the media containing 25 mM glucose (CNT), 5 mM glucose (LG), or 20 mM lactate (High lactate) for 6 h. Real-time PCR was performed using primer pairs as indicated. The mRNA levels are represented relative to the values in CNT. (**C**) A549 cells were cultured on 6 cm flat dishes were in the absence (CNT) and presence of 10 μM fasentin (Fas) or 5 mM glucose (LG) for 6 h. (**D**) A549 cells were cultured on 6 cm flat dishes (2D) and 96-well round-bottomed plates (3D). Intracellular contents of glucose and lactate were measured using each assay kit and represented relative to the values in CNT or 2D. *n* = 3–4. ** *p* < 0.01 and * *p* < 0.05 compared with CNT or 2D. NS, *p* > 0.05.

**Figure 5:**
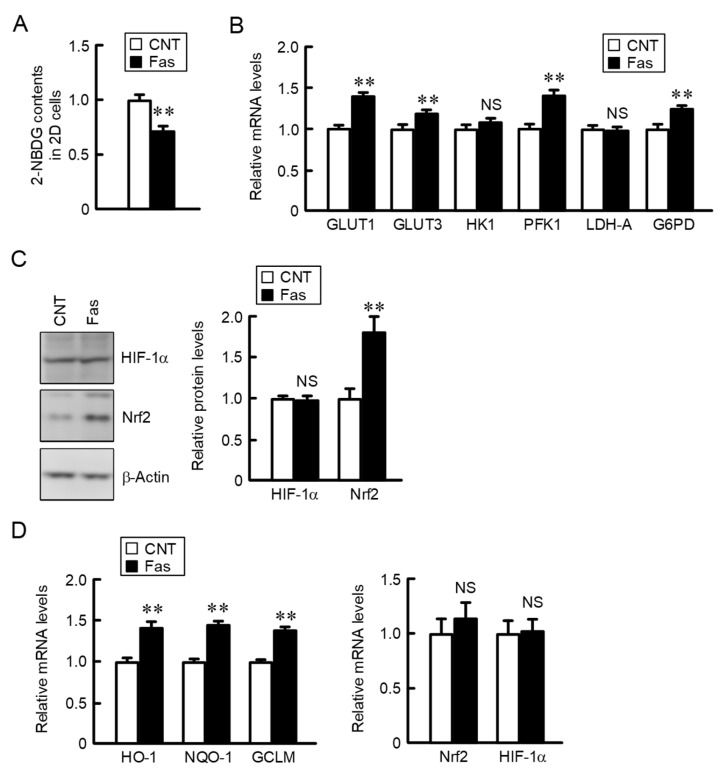
Increase in the expression of Nrf2 and Nrf2-related genes by fasentin in 2D monolayer cells. A549 cells were cultured on 96-well plates (**A**) or 6 cm dishes (**B**–**D**) for 4 days. The cells were incubated in the absence (CNT) and presence of 10 μM fasentin (Fas) for 24 h (**A**,**C**) or 6 h (**B**,**D**). (**A**) The cells were incubated with 10 μM 2-NBDG for 60 min after the treatment with Fas. The fluorescence intensity of 2-NBDG was measured using an Infinite F200 PRO microplate reader and is represented relative to the values in CNT. (**B**,**D**) Real-time PCR was performed using primer pairs as listed in Table 1. The mRNA levels are represented relative to the values in CNT. (**C**) Western blotting was performed using anti-HIF-1α, anti-Nrf2, and anti-β-actin antibodies. The protein levels are represented relative to the values in CNT. *n* = 3–4. ** *p* < 0.01 compared with CNT. NS, *p* > 0.05.

Nutrients including glucose are supplied into the cells from the blood, and the cell metabolites are released into the blood. However, there may be an insufficient exchange of nutrients and metabolites between blood vessels and the extracellular space of cancer cells because the cells of the tumor microenvironment locate away from mature blood vessels. In 2D monolayer cells, the expression of most of the glucose transporters and metabolic enzymes was increased by exposure to a low concentration of glucose (Figure 4A). In contrast, the exposure to a high concentration of lactate, an end product of anaerobic glucose metabolism, had no effect. In addition, the mRNA levels of glucose transporters and metabolic enzymes were changed by neither the exposure to low concentration of glucose nor high concentration of lactate in 3D spheroid cells (Figure 4B). The inner cells of spheroids may be continually exposed to low glucose conditions. To clarify the involvement of glucose, we investigated the intracellular content of glucose in 2D monolayer and 3D spheroid cells. The intracellular contents of glucose and lactate in 2D monolayer cells were decreased by the treatments with fasentin, a GLUT1 inhibitor, and a low glucose medium (Figure 4C). The intracellular contents of glucose and lactate in 3D spheroid cells were lower than those in 2D monolayer cells (Figure 4D). These results raise the possibility that the expression levels of glucose transporters and metabolic enzymes are regulated by the intracellular content of glucose. To clarify the hypothesis, we investigated the effect of fasentin on the expression of glucose transporters, metabolic enzymes, and Nrf2-targeted genes. The content of 2-deoxy-2-[(7-nitro-2,1,3-benzoxadiazol-4-yl)amino]-D-glucose (2-NBDG), a fluorescent marker for monitoring glucose uptake in living cells, in 2D monolayer cells was decreased by the treatment with fasentin (Figure 5A). The expression levels of most of the glucose transporters and metabolic enzymes were increased by fasentin (Figure 5B). These results are similar to those of low glucose medium. The protein level of Nrf2 was significantly increased by fasentin, but that of HIF-1α was not (Figure 5C). These results indicate that the reduction of intracellular glucose concentration may induce the activation of the Nrf2 signal. The expression levels of Nrf2-targeted genes were increased by fasentin, but those of Nrf2 and HIF-1α were not (Figure 5D). The protein level of Nrf2 may be controlled in the post-translational step as shown in spheroid cells transfected with CLDN2 siRNA (Figure 3D,E).

### 2.5. Effects of Fasentin and Low Glucose Concentration on Mitochondria Activity

**Figure 6:**
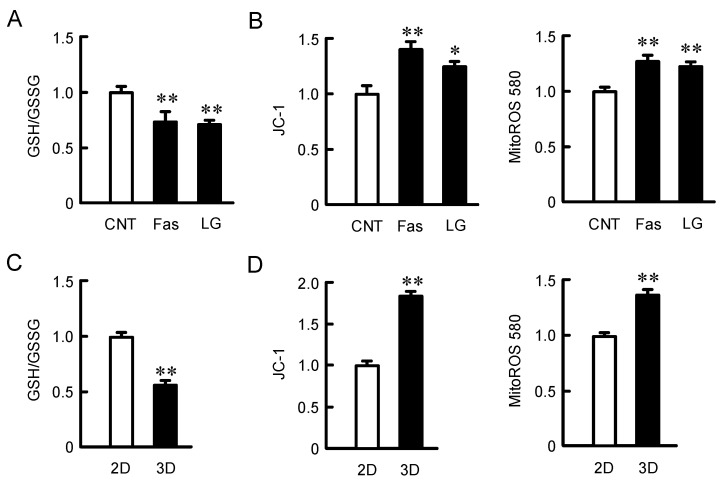
Effects of fasentin, low glucose and 3D culture on glutathione redox state and mitochondria activity. (**A**,**B**) A549 cells cultured on 96-well flat plates were incubated in the absence (CNT) and presence of 10 μM fasentin (Fas) or 5 mM glucose (LG) for 6 h. (**C**,**D**) A549 cells were cultured on 96-well flat plates (2D) or round-bottomed plates (3D). The glutathione redox state was measured by a GSSG/GSH Quantification Kit. The mitochondrial potential was examined using JC-1, and the ratios of red and green fluorescence were calculated by ImageJ. The mitochondrial ROS production was examined using MitoROS 580. The values in 3D, fasentin-treated, or LG-treated cells are represented relative to the values in CNT or 2D. *n* = 6–8. ** *p* < 0.01 and * *p* < 0.05 compared with CNT or 2D.

The expression of Nrf2 is downregulated by binding to Kelch-like ECH-associated protein 1 (Keap1), which is inhibited by oxidative stress [16]. The ratio of reduced glutathione (GSH) to oxidized glutathione (GSSG) was decreased by low glucose medium and fasentin (Figure 6A), indicating that the reduction of intracellular glucose concentration may alter the oxidation-reduction balance. Mitochondria activity and mitochondrial ROS production were monitored by JC-1 and MitoROS 580, respectively. Both the fluorescence intensity of MitoROS 580 and ratios of red to green JC-1 fluorescence intensities were increased by fasentin and low glucose medium (Figure 6B), indicating that the function of mitochondria may be enhanced by the intracellular reduction of glucose concentration. The ratio of GSH to GSSG in 3D spheroid cells was significantly lower than that in the 2D monolayer cells (Figure 6C). The fluorescence intensity of MitoROS 580 and ratios of red to green JC-1 fluorescence intensities were increased by 3D culture (Figure 6D). As shown in Figure 4C, the glucose content in the 3D spheroid cells was lower than that in the 2D monolayer cells. These results indicate that the oxidation-reduction balance in 3D cells may shift towards oxidation by the elevation of mitochondria activity and ROS production.

### 2.6. Effect of CLDN2 Knockdown on Paracellular Permeability in Monolayer Cells, Accumulation of Glucose and Oxidation-Reduction Balance in 3D Spheroid Cells

**Figure 7:**
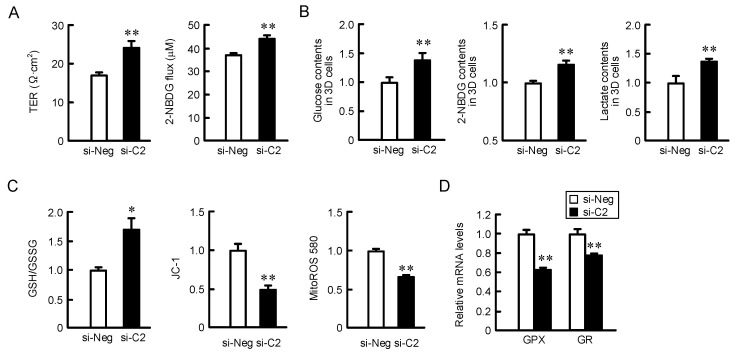
Effect of CLDN2 knockdown on paracellular permeability in 2D monolayer and accumulation in 3D spheroids against small molecules. (**A**) A549 cells transfected with negative (si-Neg) or CLDN2 siRNA (si-C2) were cultured on transwell inserts for 4 days. TER was measured using volt ohmmeter. 100 μM 2-NBDG was added on transwell inserts. After incubation at 4 °C for 60 min, the fluorescence intensity in the lower solution was measured using an Infinite F200 PRO microplate reader. (**B**) A549 cells transfected with si-Neg or si-C2 were cultured on 96-well round-bottomed plates for 4 days. The contents of glucose, 2-NBDG, and lactate were measured using each assay kit and represented to the values in si-Neg. (**C**) A549 cells transfected with si-Neg or si-C2 were cultured on 96-well round-bottomed plates for 4 days. The glutathione redox state was measured using a Kit. The mitochondrial potential and ROS production were examined using JC-1 and MitoROS 580, respectively. The values in si-C2 are represented relative to the values in si-Neg. (**D**) A549 cells transfected with si-Neg or si-C2 were cultured on 96-well round-bottomed plates for 4 days. The mRNA levels are represented relative to the values in si-Neg. *n* = 3–4. ** *p* < 0.01 and * *p* < 0.05 compared with si-Neg.

The characteristics of CLDNs expression on paracellular permeabilities of ions and small molecules are different in each CLDN subtype. So far, we have reported that CLDN2 knockdown decreases paracellular ion permeability, whereas it increases paracellular DXR permeability [14]. Similarly, CLDN2 knockdown increased paracellular permeability of 2-NBDG, although it decreased paracellular ion permeability (Figure 7A). Considering together with the results of immunofluorescence measurement and electron microscope observation, CLDN2 may form a barrier to small molecules in the outer layer of spheroids. Therefore, we decided to investigate the effect of CLDN2 knockdown on the accumulation of glucose, 2-NBDG, and lactate using spheroid cells. These contents in CLDN2-knockdown spheroid cells were higher than those in CLDN2-expressing cells (Figure 7B), indicating that CLDN2 may block the uptake of glucose and its metabolization in spheroid cells, leading to the elevation of lactate content. The ratio of GSH to GSSG was increased by CLDN2 knockdown, whereas the fluorescence intensity of MitoROS 580 and ratios of JC-1 were decreased (Figure 7C). The intracellular ROS is scavenged by not only GSH, but also glutathione peroxidase (GPX) [17]. GSSG is converted to GSH by glutathione reductase (GR). The mRNA levels of GPX and GR were decreased by CLDN2 knockdown (Figure 7D), indicating that the cells may be exposed to less oxidative stress.

### 2.7. Effects of Fasentin and Sulforaphane on DXR-Induced Cytotoxicity in 2D Monolayer and 3D Spheroid Cells

**Figure 8:**
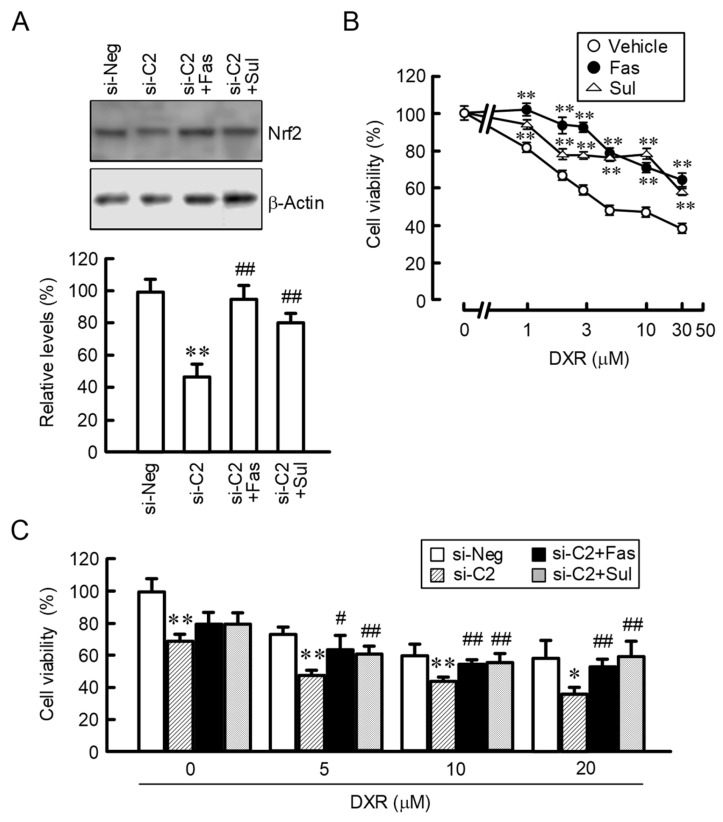
Inhibition of CLDN2 knockdown-induced chemosensitization by fasentin and sulforaphane. (**A**) A549 cells transfected with negative (si-Neg) or CLDN2 siRNA (si-C2) were cultured on 6 cm dishes for 3 days. Then, the cells were incubated in the absence and presence of 10 μM fasentin (Fas) or 10 μM sulforaphane (Sul) for 24 h. Western blotting was performed using anti-Nrf2, and anti-β-actin antibodies. The protein levels are represented relative to the values in si-Neg. (**B**) The cells transfected with si-C2 were cultured on 96-well plates for 3 days. Then, the cells were incubated in the absence (Vehicle) and presence of 10 μM Fas, 10 μM Sul, or DXR for 24 h. The cell viability was measured using the Premix WST-1 Cell Proliferation Assay System. (**C**) The cells transfected with si-Neg or si-C2 were cultured on 96 well round-bottomed plates for 3 days. Then, the cells were incubated in the absence and presence of 10 μM Fas, 10 μM Sul, or DXR for 24 h. The cell viability was measured using the CellTiter-Glo 3D Cell Viability Assay Kit. *n* = 3–4. ** *p* < 0.01 and * *p* < 0.05 compared with si-Neg or vehicle. ^##^ *p* < 0.01 and ^#^ *p* < 0.05 compared with si-C2 alone.

**Figure 9:**
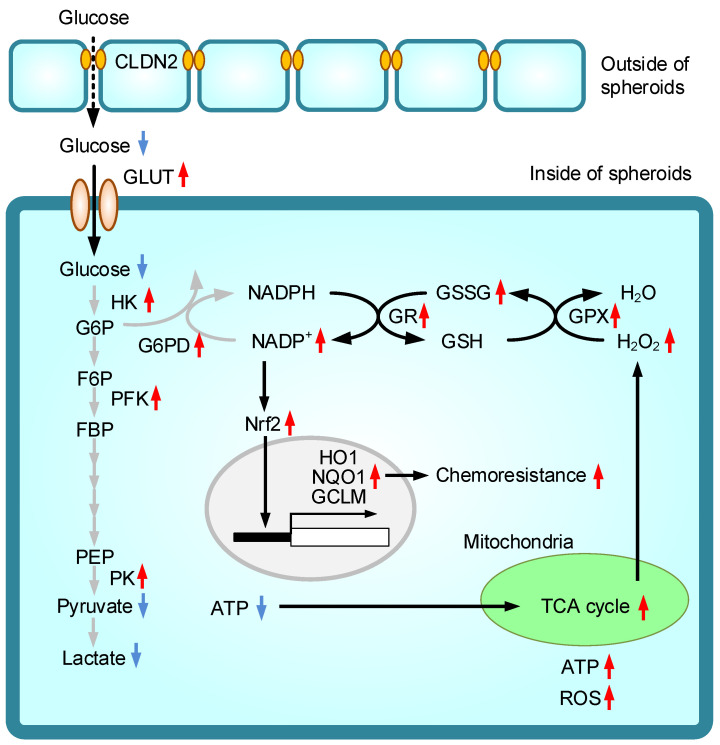
A putative model of CLDN2-induced Nrf2 signal. The cells expressing CLDN2 in the outside of spheroids block the paracellular influx of glucose. Glucose deprivation enhances the expression of glucose transporters and glucose metabolic enzymes. The reduction of glycolytic ATP production promotes the activation of mitochondrial energy metabolism and ROS generation. The oxidative stress confers chemoresistance mediated by the activation of the Nrf2 signal.

Nrf2 was reported to be involved in the chemoresistance in various cancer cells such as lung, pancreas, liver, and so on [18]. As shown in Figure 5D, the expression of Nrf2 was increased by fasentin in A549 cells. The CLDN2 knockdown-induced reduction of Nrf2 expression was restored by the treatments with fasentin and sulforaphane (Figure 8A). Therefore, we investigated the effects of fasentin and sulforaphane on the DXR-induced cytotoxicity in the 2D monolayer of CLDN2 knockdown cells. Cell viability was dose-dependently decreased by DXR and the IC_50_ was about 4.3 ± 0.5 μM in the vehicle-treated cells (Figure 8B). The chemosensitivity to DXR was decreased by the treatments with fasentin and sulforaphane, whose IC_50_ values were over 30 μM. These results raise the possibility that Nrf2 may be involved in the CLDN2-induced chemoresistance in spheroid cells. To clarify this hypothesis, we investigated the effects of fasentin and sulforaphane on the DXR-induced toxicity in spheroid cells. The cell viability was dose-dependently decreased by DXR, which was enhanced by CLDN2 knockdown (Figure 8C). The effect of CLDN2 knockdown was restored by the treatments with fasentin and sulforaphane. Similar results were obtained in PC-3 cells (Supplementary Figure S3). The putative regulatory mechanism of glucose metabolism and Nrf2 signal by CLDN2 in spheroid cells is depicted in Figure 9.

## 3. Discussion

The cancer cells in the tumor microenvironment are commonly exposed to hypoxia, oxidative, and nutritional stresses, leading to the acquisition of therapeutic resistance. So far, we reported that CLDN2, which is highly expressed in human lung adenocarcinoma tissues and cell lines, induces chemoresistance against anticancer drugs including DXR, cisplatin, and docetaxel in A549 spheroid cells [19]. Hypoxic level in spheroids is reduced about 10% by CLDN2 knockdown, suggesting that hypoxic stress may be involved in the CLDN2-induced chemoresistance. However, the level of HIF-1α, a key mediator of cellular adaptation to hypoxia, was not significantly attenuated by CLDN2 knockdown (Figure 3). Therefore, CLDN2 may confer chemoresistance to lung adenocarcinoma cells mediated through other mechanisms.

Abnormal expression of CLDNs including CLDN2 was reported in various solid tumors [20]. The pathophysiological function of each CLDN varies among different tissue types. In many cases, the downregulation of CLDN subtypes expression contributes to epithelial-mesenchymal transition. In contrast, upregulation of CLDN subtypes expression promotes the abilities of proliferation and metastasis. There are few reports showing that CLDN functions as a paracellular barrier to small molecules. So far, we reported that CLDN2 may suppress paracellular permeabilities to small molecules such as DXR and lucifer yellow, a water-soluble fluorescent dye [14]. In transwell assay, CLDN2 knockdown increased TER, which means CLDN2 forms a pore to ions, whereas it increased paracellular permeability to 2-NBDG, a fluorescent deoxyglucose derivative (Figure 7A). In addition, the accumulation of glucose and 2-NBDG in spheroid cells was enhanced by CLDN2 knockdown (Figure 7B). These results suggest that CLDN2 plays a role as a barrier to small molecules in A549 spheroid cells.

The glycolysis pathway enables cancer cells to avoid excess ROS generation from mitochondrial respiration. The reduction of ROS generation by electron transport chain enzymes in the mitochondria is accompanied with downregulation of mitochondrial ATP synthesis. Cancer cells utilize the anaerobic glycolysis pathway to rapidly produce ATP, which is required for the high metabolic demands of the cancer cells. The expression levels of glucose transporters and metabolic enzymes were increased by fasentin and low glucose medium in 2D monolayer cells (Figure 4 and Figure 5). These expression levels were decreased by CLDN2 knockdown in spheroid cells, suggesting that CLDN2 is involved in the regulation of the glycolysis pathway. Although the expression levels of glucose metabolic enzymes were high in spheroid cells, the lactate content in spheroid cells was lower than that in 2D monolayer cells (Figure 4C). Similarly, the lactate content was not shifted in proportion to glucose metabolic enzymes by fasentin and low glucose medium in 2D monolayer cells (Figure 4 and Figure 5). CLDN2 may inhibit ATP production in the glycolysis pathway mediated by the block of glucose influx in spite of the elevation of glucose metabolic enzymes. Anti-glycolytic agents such as fasentin, 2-deoxy-D-glucose, and 3-bromopyruvate, which can shift the glucose metabolic pathway from glycolysis to the more conventional mitochondrial oxidative phosphorylation, were reported to be useful in preclinical and clinical development for anticancer therapy [21]. Despite that, mitochondria play important roles in the regulation of proliferation and survival in cancer cells mediated through the maintenance of oxidative equilibrium, mitochondrial membrane potential, and the productions of lipids, amino acids, and nucleotides [22]. Metformin, a complex I inhibitor used in the treatment of diabetes, has anticancer activity in diabetics [23]. In contrast, the elevation of mitochondrial ROS production, which facilitates cancer cell death, may enhance the activity of chemotherapy [24]. Our results indicate that mitochondrial membrane potential and ROS production were increased by glucose deprivation and fasentin in 2D monolayer cells (Figure 6), whereas they were decreased by CLDN2 knockdown in spheroid cells (Figure 7C). The DXR-induced toxicity was enhanced by CLDN2 knockdown in spheroid cells (Figure 8). Although the reduction of CLDN2 expression promotes the delivery of glucose into spheroid cells, the sensitivity to anticancer drugs may be attenuated by the reduction of mitochondria activity and ROS production. In the future study, we need to investigate the involvement of glucose catabolism on CLDN2-induced change of mitochondria activity.

The expression of Nrf2 in spheroid cells was higher than that in 2D monolayer cells (Figure 2), and the expression was attenuated by CLDN2 knockdown (Figure 3). The protein expression of Nrf2 is mainly controlled by Keap1-mediated ubiquitination and the proteasomal degradation system [25]. Although the protein level of Nrf2 was reduced by CLDN2 knockdown in spheroid cells, the mRNA level of Nrf2 was unchanged (Figure 3D,E), suggesting that CLDN2 may suppress the degradation of Nrf2 protein. The activation of the Nrf2 signal is one of the major defense systems that facilitate cell survival under toxic conditions. Low expression or inactivated mutation of Keap1 occurs in human non-small-cell lung carcinoma tissues including adenocarcinoma, leading to the increased expression and activation of Nrf2 [26,27]. The expression of Nrf2 was reported to be correlated with mitochondrial redox status [28]. Our results indicated that fasentin and glucose deprivation increase the mitochondrial membrane potential and ROS production in 2D monolayer cells (Figure 6). In addition, the mitochondrial membrane potential and ROS production in spheroid cells were decreased by CLDN2 knockdown (Figure 7). Therefore, CLDN2 may activate the Nrf2 signal in A549 spheroid cells mediated through the elevation of mitochondrial ROS production.

The CLDN2 knockdown enhanced sensitivity to DXR in spheroid cells, but the mechanisms have not been clarified in detail. We previously reported that CLDN2 may block the influx of DXR into spheroid cells [14]. Here, we found that CLDN2 knockdown attenuated the expression of Nrf2 and the Nrf2-targeted genes (Figure 3D,E). The DXR-induced toxicity was enhanced by CLDN2 knockdown, which was inhibited by an Nrf2 activator sulforaphane (Figure 8C). Therefore, Nrf2-mediated antioxidant response may be also involved in the CLDN2-induced chemoresistance. Cancer stem cells, which comprise a small subpopulation of cancerous cells, have exclusive abilities in self-renewing, and stemness, drug resistance. The Nrf2 activation increases the proportions of cancer stem cells in breast cancer cells [29]. CLDN2 may be involved in the regulation of cancer stem cells in A549 spheroids. Another explanation is that glucose deprivation induces AMPK activation and autophagy [30,31]. The autophagy-induced chemoresistance is reported in various solid tumors including lung adenocarcinoma [32]. We need further study to clarify the mechanism underlying CLDN2-induced chemoresistance in lung adenocarcinoma.

## 4. Materials and Methods

### 4.1. Cell Culture

Human lung adenocarcinoma-derived A549 cells were purchased from the RIKEN BRC through the National Bio-Resource Project of the MEXT (Ibaraki, Japan). The cells were maintained in Dulbecco’s modified Eagle’s medium (Sigma-Aldrich, Saint Louis, MO, USA) as described previously [33]. The cell viabilities in 2D monolayer, which cultured on 96-well flat-bottomed plates (Thermo Fisher Scientific, Rochester, NY, USA), and 3D spheroids, which cultured on 96-well round-bottomed plates (Sumilon, Sumitomo, Osaka, Japan), were examined using the Premix WST-1 Cell Proliferation Assay System (Takara-Bio, Shiga, Japan) and CellTiter-Glo 3D Cell Viability Assay Kit (Promega, Madison, WI, USA), respectively, according to the manufacturer’s instructions. In the assay of chemosensitivity to DXR (Fujifilm Wako Pure Chemical, Osaka, Japan), the spheroid cells were treated with DXR for 24 h.

### 4.2. Reverse Transcription and Quantitative Real-Time PCR

Total RNA was isolated from 2D monolayer and 3D spheroid cells using TRI reagent (Molecular Research Center, Cincinnati, OH, USA). Reverse transcription and quantitative real-time PCR were performed using ReverTra Ace Kit and THUNDERBIRD qPCR Mix (Toyobo, Osaka, Japan), respectively. The specific primers against human glucose transporters, glucose metabolic enzymes, oxidative stress markers, and CLDN2 are listed in Table 1. The relative change in mRNA expression was calculated as described previously [34].

### 4.3. Sodium Dodecyl Sulfate-Polyacrylamide Gel Electrophoresis and Western Blotting

The cells cultured on 6-well plates and 96-well round-bottomed plates were collected by centrifugation. The preparation of cell lysates and Western blotting were performed as described previously [33]. Primary antibodies used in Western blotting and immunocytochemistry were listed in Table 2. The blots were scanned using a C-DiGit Blot Scanner (LI-COR Biotechnology, Lincoln, NE, USA). Band density was quantified using ImageJ software 1.53J (National Institute of Health, Bethesda, MD, USA). The signals were normalized to β-actin, an internal loading control.

### 4.4. Microarray Analysis

The cells cultured on 96-well round-bottomed plates were transfected with negative or CLDN2 siRNA using ScreenFect A (Fujifilm Wako Pure Chemical). After 96 h of transfection, total RNA was isolated from the cells using NucleoSpin RNA (Takara-Bio). Microarray analysis was performed using Clariom S Assay for humans (Filgen, Nagoya, Japan). The expression levels of 41 genes were upregulated by 2-fold or greater, whereas 108 genes were downregulated by half or less (Table 3).

### 4.5. Immunocytochemistry

Spheroid cells transfected with negative or CLDN2 siRNA were fixed with 3.7% formaldehyde for 30 min at room temperature, followed by treating with CytoVista 3D Cell Culture Clearing/Staining Kit (Thermo Fisher Scientific). Then the cells were permeabilized with 0.2% Triton X-100 for 15 min, followed by blocking with 4% Block Ace (Dainippon Sumitomo Pharma, Osaka, Japan) for 30 min. The cells were incubated with the anti-CLDN2 antibody for 16 h at 4 °C, and then incubated with Alexa Fluor 555-conjugated secondary antibody plus DAPI for 1.5 h at room temperature. The localization of CLDN2 was visualized using an LSM 700 confocal microscope (Carl Zeiss, Jena, Germany).

### 4.6. Electron Microscopy

Spheroid cells transfected with negative or CLDN2 siRNA were fixed with 2% paraformaldehyde and 2% glutaraldehyde in 0.1 M phosphate buffer (PB) at 4 °C overnight. The preparation and electron microscopic analysis of samples were performed by Tokai Electron Microscopy (Nagoya, Japan). The fixed samples were postfixed with 2% osmium tetroxide in 0.1 M PB at 4 °C for 2 h, followed by dehydration, infiltration, and polymerization. Then, the polymerized resins were ultra-thin sectioned at 70 nm with a diamond knife using an ultramicrotome (Ultracut UCT, Leica, Vienna, Austria) and the sections were mounted on copper grids. They were stained with 2% uranyl acetate at room temperature for 15 min and then washed with distilled water followed by being secondary-stained with Lead stain solution (Sigma-Aldrich) at room temperature for 3 min. The grids were observed by a transmission electron microscope (JEM-1400Plus, JEOL, Tokyo, Japan) at an acceleration voltage of 100 kV. Digital images were taken with a CCD camera (EM-14830RUBY2, JEOL).

### 4.7. Intracellular Glutathione, Glucose and Lactate Contents

The intracellular contents of glutathione, glucose, and lactate were measured using GSSG/GSH Quantification Kit, Glucose Assay Kit-WST, and Lactate Assay Kit-WST (Dojindo Laboratories, Kumamoto, Japan), respectively, according to the manufacturer’s instructions.

### 4.8. Mitochondrial ROS Production and Membrane Potential

Cells cultured on 96-well plates were treated with 25 μM fasentin or glucose-free medium for 6 h. Then, the cells were incubated with MitoROS 580 (AAT Bioquest, Sunnyvale, CA, USA) or JC-1 iodide (Takara-Bio) at 37 °C for 30 min. After washing twice with Hank’s balanced salt solution, the fluorescence images were observed using BZ-X810 (Keyence, Osaka, Japan). The fluorescence intensity of MitoROS 580 was represented as a percentage of control. In the case of JC-1, the ratios of red to green JC-1 fluorescence intensities were calculated, followed by representing as a percentage of control.

### 4.9. Measurement of Barrier Function of TJs

Cells were cultured on transwell plates (0.4 μm pore size, 12 mm diameter) with polyester membrane inserts (Corning Incorporated, Corning, NY, USA). The barrier function of TJs was estimated by TER and paracellular flux of 2-NBDG. TER was measured using volt ohmmeter [33,35]. In the 2-NBDG flux assay, 100 μM 2-NBDG was added to transwell inserts. After incubation at 4 °C for 30 or 60 min, the solution in the lower compartment was collected and the fluorescence intensity of 2-NBDG was measured using an Infinite F200 PRO microplate reader (Tecan, Mannedorf, Switzerland). The concentration of 2-NBDG was calculated using a calibration curve.

### 4.10. Statistical Analysis

Results are presented as means ± S.E.M. Each study was repeated at least three times. Differences between groups were analyzed by one-way analysis of variance, and corrections for multiple comparisons were made using Tukey’s multiple comparison test. Comparisons between two groups were made using Student’s *t*-test. Statistical analyses were performed using KaleidaGraph version 4.5.1 software (Synergy Software, Reading, PA, USA). Significant differences were assumed at *p* < 0.05.

## 5. Conclusions

We found that CLDN2 induces the acquisition of chemoresistance in A549 spheroid cells mediated by the activation of the Nrf2 signal. The expression of the glucose transporter and metabolic enzymes were decreased by the CLDN2 knockdown. The expression of Nrf2 was upregulated by fasentin and glucose deprivation in 2D monolayer cells. The glucose content in spheroids was increased by the CLDN2 knockdown. These results indicate that CLDN2 can play an important role in the regulation of glucose influx, glucose metabolization and the Nrf2 signal in A549 spheroid cells.

## Figures and Tables

**Table 1:** Primer pairs for real-time PCR.

Genes	Direction	Sequence (5′-----3′)
*GLUT1*	Sense	CAGCTACCCTGGATGTCCTATC
	Antisense	AATTTGAGGTCCAGTTGGAGAA
*GLUT3*	Sense	GCTGCTACTGGGTTTTACCATC
	Antisense	CTTGTGACATCCTTGCACTCTC
*HK1*	Sense	GAAAAAGAGAACGGTGGAAATG
	Antisense	GTCAGAGATGCAGGAGACAATG
*PFK1*	Sense	CTTGTCACCTCTCTGTCCTGTG
	Antisense	ACAGTAACCCGGGTGTCATATC
*PK1*	Sense	TGGGTCTCTAAGTCCCAAAGAG
	Antisense	AATGTCCAGTAGGCAGAGGTGT
*LDH-A*	Sense	GGAGTTCACCCATTAAGCTGTC
	Antisense	GTGAACCTCTTTCCACTGTTCC
*G6PD*	Sense	CGTGTACACCAAGATGATGACC
	Antisense	GGGAGCTTCACGTTCTTGTATC
*HO-1*	Sense	AAGATTGCCCAGAAAGCCCTGGAC
	Antisense	AACTGTCGCCACCAGAAAGCTGAG
*NQO-1*	Sense	GAAGAGCACTGATCGTACTGGC
	Antisense	GGATACTGAAAGTTCGCAGGG
*GCLM*	Sense	TGTCTTGGAATGCACTGTATCTC
	Antisense	CCCAGTAAGGCTGTAAATGCTC
*GPX1*	Sense	TGCGGGGCAAGGTACTACTTAT
	Antisense	GGACGTACTTGAGGGAATTCAG
*GR*	Sense	GAGCTTTACCCCGATGTATCAC
	Antisense	GACTGTGTTGTCAAAGTCTGCC
*CLDN2*	Sense	ATTGTGACAGCAGTTGGCTT
	Antisense	CTATAGATGTCACACTGGGTGATG
*β-Actin*	Sense	CCTGAGGCACTCTTCCAGCCTT
	Antisense	TGCGGATGTCCACGTCACACTTC

**Table 2:** Primary antibodies for Western blotting and immunocytochemistry.

Name	Catalog Number	Supplier	Address
GLUT1	10-7548	Abeomics	San Diego, CA, USA
GLUT3	20403-1-AP	ProteinTech	Rosemont, IL, USA
HK1	200-4159S	Rockland Immunochemicals	Limerick, PA, USA
PFK1	200-1156S	Rockland Immunochemicals	Limerick, PA, USA
PK1	200-1178S	Rockland Immunochemicals	Limerick, PA, USA
LDH-A	sc-137243	Santa Cruz Biotechnology	Santa Cruz, CA, USA
G6PD	25413-1-AP	ProteinTech	Rosemont, IL, USA
β-Actin	sc-1615	Santa Cruz Biotechnology	Santa Cruz, CA, USA
HIF-1α	bs-0737R	Bioss Antibodies	Woburn, MA, USA
Nrf2	GTX102572	GeneTex	Irvine, CA, USA
CLDN2	51-6100	Thermo Fisher Scientific	Rockford, IL, USA

**Table 3:** List of genes upregulated or downregulated by CLDN2 knockdown.

2-Fold or Greater		Half or Less		
*EPB41*	*CTAG1B*	*AGPAT1*	*GNB2*	*PTOV1*
*LGALS8*	*KRTAP5-6*	*ANKRD54*	*GOLT1A*	*RFXANK*
*SV2A*	*MRGPRD*	*ANKZF1*	*GPX1*	*RGL2*
*LPGAT1*	*SYTL2*	*AP4M1*	*HINFP*	*RHBDD2*
*PLD5*	*IL18*	*APOL2*	*HIST1H2AB*	*RNASEH2A*
*TNFRSF25*	*LYRM5*	*ASIC1*	*HMOX1*	*RNF123*
*GCKR*	*UBE3B*	*BDKRB2*	*HPCAL1*	*RTN4RL2*
*C2orf71*	*FGF6*	*C20orf96*	*HSD17B14*	*S100A13*
*CCNL1*	*KRT6B*	*CA9*	*HSPBP1*	*SERINC2*
*PARM1*	*KCNA6*	*CCDC102A*	*ID3*	*SIPA1*
*GAR1*	*LINC00452*	*CCM2*	*ILVBL*	*SLC16A3*
*TRIM2*	*SAMD4A*	*CD151*	*KCNK5*	*SLC29A4*
*TMEM167A*	*TMOD3*	*CDC42EP4*	*LAMTOR1*	*SLX1A*
*CLPS*	*ADAMTS17*	*CDH16*	*LIMK1*	*SPAG4*
*DLD*	*COL1A1*	*CFD*	*LINC01588*	*SPNS1*
*CSPP1*	*SEZ6*	*CLDN2*	*LTBR*	*SRXN1*
*BRF2*	*SPIRE1*	*COX5B*	*LYPLA2*	*SUMF1*
*JAK2*	*TCF4*	*COX7A2*	*MCM5*	*TBC1D16*
*IL11RA*	*ANGPTL8*	*CRIP1*	*MIF*	*TCEB2*
*PGRMC1*	*ZNF253*	*CUEDC2*	*MIF4GD*	*TEX264*
		*CYB5R1*	*MRPS12*	*TIMM13*
		*DEDD2*	*MYL6B*	*TMEM205*
		*DNAL4*	*NDRG1*	*TMEM219*
		*ECI1*	*NDUFA13*	*TMEM222*
		*EFNA1*	*NT5C*	*TMEM8A*
		*EIF4EBP1*	*OTUD7B*	*TNFRSF12A*
		*EIF6*	*PFKFB4*	*TPI1*
		*ETFB*	*PFKL*	*TPRA1*
		*EXOC7*	*PGAP2*	*TRAF4*
		*FAM134C*	*PLPPR2*	*TRAPPC5*
		*FAM96B*	*PMM1*	*TSC22D4*
		*FIBP*	*POLE4*	*UQCRC1*
		*FIZ1*	*PQBP1*	*VPS28*
		*FLOT1*	*PRDX5*	*ZMYM3*
		*GANC*	*PREB*	*ZNF358*
		*GC*	*PSMB5*	*ZNF428*

## Data Availability

All data generated or analyzed during this study are included in this published article.

## Supplementary Materials

The following are available online at https://www.mdpi.com/article/10.3390/ijms22126582/s1.

## Abbreviations

ATP	adenosine tri-phosphate
CLDN	claudin
DAPI	4′6-diamidino-2-phenylindole
DXR	doxorubicin
3D	three-dimensional
GCLM	glutamate-cysteine ligase modifier subunit
G6PD	glucose-6-phosphate dehydrogenase
GLUT	sodium-independent glucose transporter
GPX	glutathione peroxidase
GR	glutathione reductase
GSH	reduced glutathione
GSSG	oxidized glutathione
HIF-1	hypoxia-inducible factor-1
HK1	hexokinase 1
HO-1	heme oxygenase-1
Keap1	Kelch-like ECH-associated protein
LDH-A	lactate dehydrogenase-A
2-NBDG	2-deoxy-2-[(7-nitro-2,1,3-benzoxadiazol-4-yl)amino]-D-glucose
NQO1	NAD(P)H: quinone oxidoreductase-1
NRF2	nuclear factor erythroid 2-related factor 2
PCR	polymerase chain reaction
PFK1	phosphofructokinase 1
PFKFB4	6-phosphofructo-2-kinase/Fructose-2,6-biphosphatase 4
PFKL	6-phosphofructokinase
PK1	pyruvate kinase 1
ROS	reactive oxygen species
SGLT	sodium-dependent glucose transporter
TER	transepithelial electrical resistance
TJ	tight junction
