# Supplementary material for: Elevation of Chemosensitivity of Lung Adenocarcinoma A549 Spheroid Cells by Claudin-2 Knockdown through Activation of Glucose Transport and Inhibition of Nrf2 Signal
Source: Int J Mol Sci. 2021 Jun 19;22(12):6582. doi: 10.3390/ijms22126582 (PMC8235168; doi:10.3390/ijms22126582)
Supplement: Supplementary file 1 [file ijms-22-06582-s001.zip › ijms-1249513-supplementary.pdf]

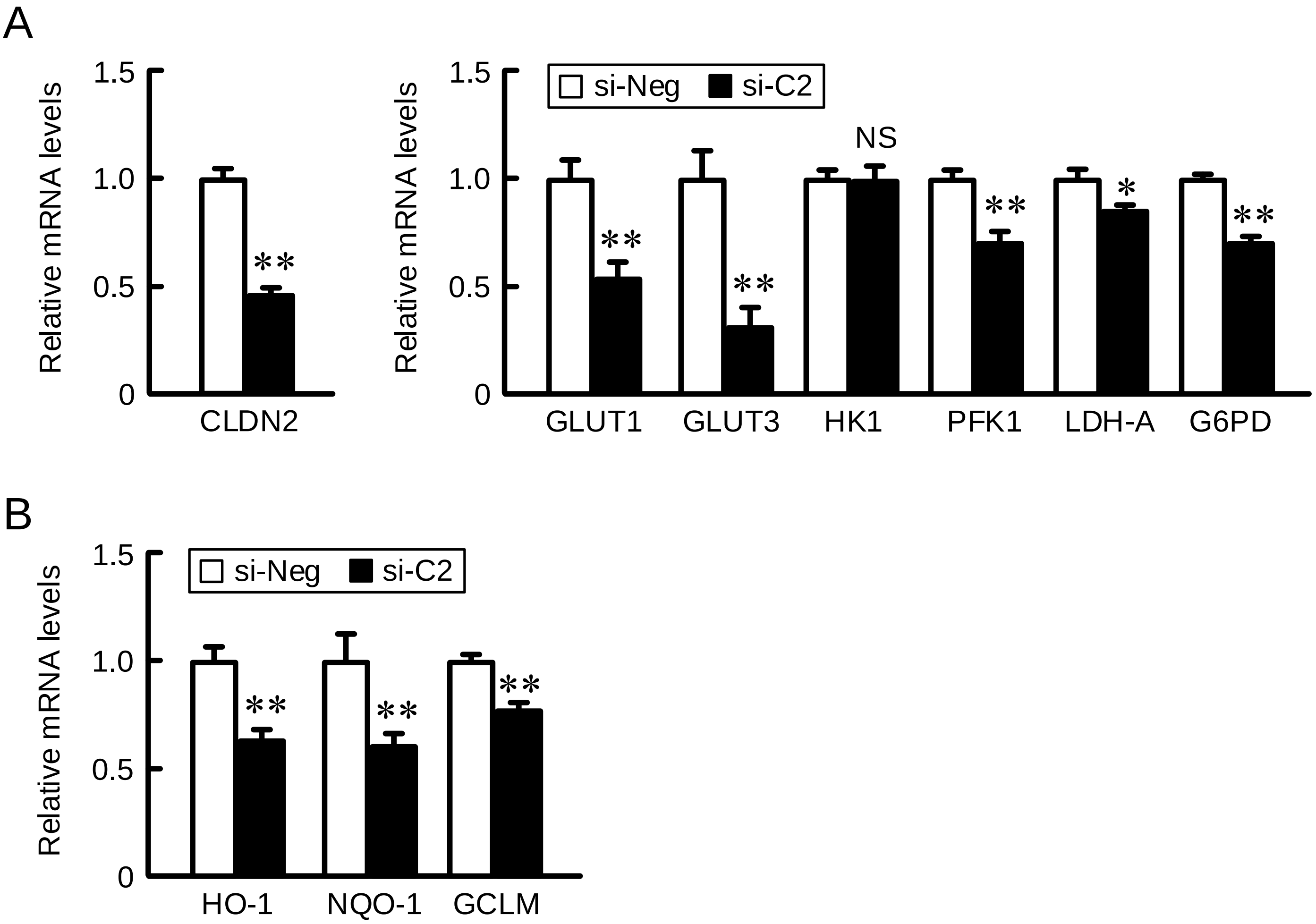

Supplementary figure S1. Decrease in glucose transporters and metabolic enzymes by CLDN2 knockdown in PC-3 spheroid cells. PC-3 cells transfected with negative (si-Neg) or CLDN2 siRNA (si-C2) were cultured on 96 well round-bottomed plates for 4 days. Real-time PCR was performed using primer pairs for CLDN2, glucose transporters, glucose metabolic enzymes (A), and Nrf2-related genes (B). The mRNA levels are represented relative to the values in si-Neg. ** $P < 0.01$ and * $P < 0.05$ compared with si-Neg. NS $P > 0.05$.

Supplementary figure S2

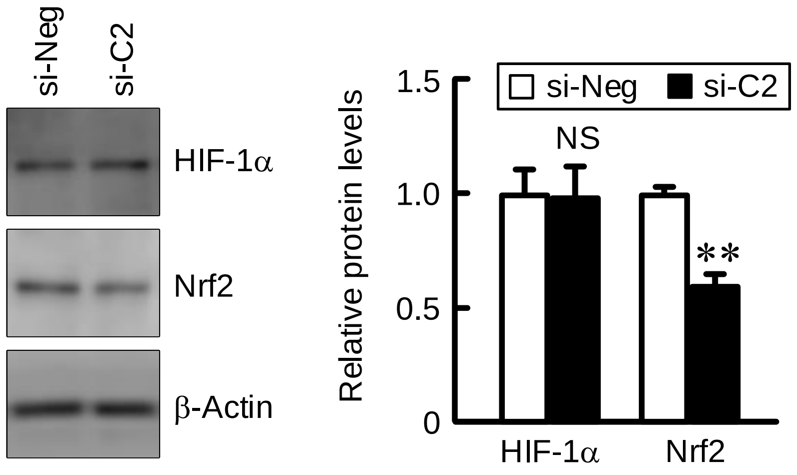

Supplementary figure S2. Decrease in Nrf2 expression by CLDN2 knockdown in PC-3 spheroid cells. PC-3 cells transfected with negative (si-Neg) or CLDN2 siRNA (si-C2) were cultured on 96 well round-bottomed plates for 4 days. Western blotting was performed using anti-HIF-1α, anti-Nrf2, and anti-β-actin antibodies. The protein levels are represented relative to the values in si-Neg. ** $P < 0.01$ compared with si-Neg. NS $P > 0.05$.

Supplementary figure S3

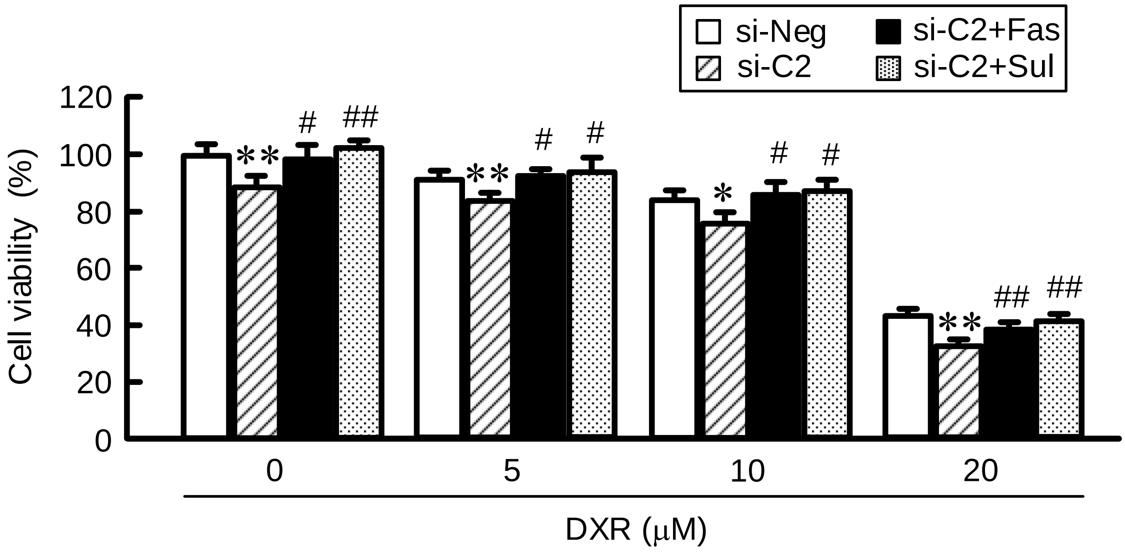

Supplementary figure S3. Inhibition of CLDN2 knockdown-induced chemosensitization by fasentin and sulforaphane. PC-3 cells transfected with negative (si-Neg) or CLDN2 siRNA (si-C2) were cultured on 96 well round-bottomed plates for 3 days. Then, the cells were incubated in the absence and presence of 10 μM fasentin (Fas), 10 μM sulforaphane (Sul), or doxorubicin (DXR) for 24 h. The cell viability was measured using the CellTiter-Glo 3D Cell Viability Assay Kit. n = 3-4. ** $P < 0.01$ and * $P < 0.05$ compared with si-Neg. $^{\#\#}$ $P < 0.01$ and $^{\#}$ $P < 0.05$ compared with si-C2 alone.
